# Mettl1-mediated internal m^7^G methylation of Sptbn2 mRNA elicits neurogenesis and anti-alzheimer’s disease

**DOI:** 10.1186/s13578-023-01131-2

**Published:** 2023-10-01

**Authors:** Qingfeng Li, Hui Liu, Lishi Li, Haomin Guo, Zhihao Xie, Xuejian Kong, Jiamin Xu, Junlin Zhang, Yunxia Chen, Zhongsheng Zhang, Jun Liu, Aiguo Xuan

**Affiliations:** 1 The Sixth Affiliated Hospital of Guangzhou Medical University, Qingyuan People’s Hospital, Qingyuan, 511518 China; 2 School of Basic Medical Sciences, First Clinical School, School of Health Management, Guangzhou Medical University, Guangzhou, 511436 China; 3 Department of Neurology, Institute of Neuroscience, Key Laboratory of Neurogenetics and Channelopathies of Guangdong Province and the Ministry of Education of China, The Second Affiliated Hospital of Guangzhou Medical University, Guangzhou, 510260 China; 4 School of Basic Medical Sciences of Guangzhou Medical University, Guangzhou Municipal and Guangdong Provincial Key Laboratory of Protein Modification and Degradation, Guangzhou, 511436 China

**Keywords:** Alzheimer’s disease, Neurogenesis, 7-Methylguanosine, Mettl1, Sptbn2

## Abstract

**Background:**

N^7^-methylguanosine (m^7^G) is one of the most conserved modifications in nucleosides impacting mRNA export, splicing, and translation. However, the precise function and molecular mechanism of internal mRNA m^7^G methylation in adult hippocampal neurogenesis and neurogenesis-related Alzheimer’s disease (AD) remain unknown.

**Results:**

We profiled the dynamic Mettl1/Wdr4 expressions and m^7^G modification during neuronal differentiation of neural stem cells (NSCs) in vitro and in vivo. Adult hippocampal neurogenesis and its molecular mechanisms were examined by morphology, biochemical methods and biological sequencing. The translation efficiency of mRNA was detected by polysome profiling. The stability of Sptbn2 mRNA was constructed by RNA stability assay. APPswe/PS1ΔE9 (APP/PS1) double transgenic mice were used as model of AD. Morris water maze was used to detect the cognitive function.

**Methods:**

We found that m^7^G methyltransferase complex Mettl1/Wdr4 as well as m^7^G was significantly elevated in neurons. Functionally, silencing Mettl1 in neural stem cells (NSCs) markedly decreased m^7^G modification, neuronal genesis and proliferation in addition to increasing gliogenesis, while forced expression of Mettl1 facilitated neuronal differentiation and proliferation. Mechanistically, the m^7^G modification of Sptbn2 mRNA by Mettl1 enhanced its stability and translation, which promoted neurogenesis. Importantly, genetic defciency of Mettl1 reduced hippocampal neurogenesis and spatial memory in the adult mice. Furthermore, Mettl1 overexpression in the hippocampus of APP/PS1 mice rescued neurogenesis and behavioral defects.

**Conclusion:**

Our findings unravel the pivotal role of internal mRNA m^7^G modification in Sptbn2-mediated neurogenesis, and highlight Mettl3 regulation of neurogenesis as a novel therapeutic target in AD treatment.

**Supplementary Information:**

The online version contains supplementary material available at 10.1186/s13578-023-01131-2.

## Introduction

Alzheimer’s disease (AD) is the most common irreversible type of dementia characterized by progressive memory loss and cognitive dysfunction together with a particular neuropathology including neuronal loss, amyloid beta (Aβ) deposition and neurofibrillary tangles [1]. AD prevalence is rapidly increasing in the aging society and contributes to substantial medical and economic burden. However, current treatments are still unable to achieve satisfactory therapeutic effects or prevent the progression of AD. Thus, it is urgent to clarify the molecular mechanisms of AD and identify potential therapeutic targets for the prevention and treatment of AD. Cumulative data reveal that adult hippocampal neurogenesis (AHN) is impaired and could result in cognitive impairment and the marked neuronal loss in patients with AD [2, 3]. Thus, in-depth understanding of the molecular mechanisms underlying AHN becomes particularly crucial when considering AHN as a therapeutic target for AD.

N^7^-methylguanosine (m^7^G) is one of the most common modifications occurring in tRNA, rRNA, mRNA cap and internal mRNA, and evolutionarily well-conserved among prokaryotes, eukaryotes and some archaea [4, 5]. The m^7^G methylation catalyzed by the methyltransferase-like 1 (Mettl1) and WD repeat domain 4 (Wdr4) complex in mammals modulates various biological processes of the mRNA life cycle [5, 6]. Internal m^7^G methylation within mRNA installed by Mettl1/Wdr4 accelerates translation efficiency of m^7^G-Modified Transcripts [5, 7]. Recent study highlights the critical role of Mettl1-mediated m^7^G tRNA modification in the self-renewal, pluripotency, and differentiation of mouse embryonic stem cells (mESCs) through regulating mRNA translation, suggesting molecular evidence of m^7^G tRNA modification in human developmental diseases [4]. Mettl1-mediated m^7^G methylation modulates the pluripotency and differentiation of human-induced pluripotent stem cells (hiPSCs), implying its potential roles in vascular development and the treatment of vascular diseases [8, 9]. Furthermore, the Disruptions of internal m^7^G and its catalyzing enzymes are associated with many diseases including microcephalic primordial dwarfism, Galloway-Mowat syndrome [10, 11]. Although its functional importance in stem cell biology and development, the precise role and regulatory mechanism of Mettl1-mediated m^7^G modification within mRNA in neurogenesis and neurogenesis-related AD remain elusive.

Here we found that m^7^G and its catalytic enzymes Mettl1/Wdr4 were markedly up-regulated in neurons and promoted neurogenesis in vitro and in vivo. Mechanistically, Mettl1-mediated m^7^G modification within mRNA enhanced the stability and translation of Sptbn2 mRNA. In addition, overexpressing Mettl1 notably rescued the reduced hippocampal neurogenesis and behavioral defects in APP/PS1 mice. Our data uncover the biological function and the underlying molecular mechanism of m^7^G modification installed by Mettl1 in hippocampal neurogenesis, providing the novel therapeutic targets for AD.

## Methods and materials

### Cell culture and transfection

Adult hippocampal neural stem cells (NSCs) were purchased from Merck-Millipore (SCR022). The NSCs proliferated in the neural stem cell basal medium (SCM003, Millipore) with fibroblast growth factor 2 (FGF2, 20 ng/mL, Millipore) on the dishes coated with 50 µg/ml poly-L-ornithine and 10 µg/ml fibronectin (Sigma-Aldrich). To induce neuron-specific differentiation, the basal medium needed to add 1 µM retinoic acid (Sigma-Aldrich) plus 5 µM forskolin (Sigma-Aldrich), and to induce astrocyte-specific differentiation, the basal medium needed to add 50 ng/ml LIF (Merck Millipore) plus 50 ng/ml BMP-2 (R&D Systems). Both of them needed to maintain for 7 days.

The plasmid construction and lentivirus packaging of this experiment was provided by OBiO Technology (Shanghai) Co., Ltd. These were the target sequences of short hairpin RNAs (shRNAs) : 1-Sh-Mettl1, 5′-GGTGAAGGTGTCCGACTAT-3′; 2-Sh-Mettl1, 5′-GACCCACACTTTAAGCGAA-3′; 1-Sh-Sptbn2, 5′-GCTGGTGTCCAAGGGCAATAT-3′; 2-Sh-Sptbn2, 5′-GCAGCCAGCAGTCAAGATATG-3′. For Mettl1 overexpression, the sequences encoding Mettl1 was inserted into the lentivirus which consisted of pSLenti-EF1-EGFP-F2A-Puro-CMV-Mettl1-WPRE. Due to the quite long sequences of Sptbn2, CRISPR/Cas9 system was used into lentivirus vector expressing Sptbn2, designing single guide RNAs (sgRNAs) whose sequence was CTCAGGAGTATATAACCCAA. After preparing all lentivirus, the optimal virus concentration was determined for transfecting cells by pre-experiment. Stable knockdown and overexpression lines were generated by lentivirus transfection and puromycin selection (1 µg/ml).

### Animals and virus injection

Male C57BL/6 mice and APP/PS1 (B6C3-Tg (APPswe, PS1dE9) 85Dbo/J) mice (28 weeks old, weighing 27–36 g, SYXK-2021-0168), 3–5 per cage, were given free access to standard food and water, and were housed under standard laboratory conditions. The mice were acclimated to laboratory prior to the testing. The 24 male C57BL/6 mice were randomized into Vector (Control group) and shRNA Mettl1 group. The 16 APP/PS1 mice were randomized into 2 groups: (1) APP/PS1 mice + Vector; (2) APP/PS1 mice + Overexpressing Mettl1. All experiments were performed in a blinded fashion. The experiments were conducted in accordance with the “Guidelines for the Protection and Use of Animals in China”, and the experimental protocols were approved by the Animal Ethics Committee of Guangzhou Medical University.

Mettl1-specific shRNA (TargetSeq1 CCCACACTTTAAGCGAACGAA, Target Seq2 GCCATGAAACACCTTCCTAAT) retroviral particles and retrovirus overexpressing Mettl1 were obtained from OBiO Technology (Shanghai) Co., Ltd. 1 µl of retroviral solution with a titer of 2☓10^8^ units/ml was injected at a rate of 0.1 µl/min into the bilateral hippocampal dentate gyrus (2.0 mm posterior to the bregma, ± 1.5 mm lateral to the midline, 2.0 mm deep from the top of the skull). After viral injections, the needle was held still for 10 min and then slowly withdrawn. The skin was closed with absorbable sutures. The mice were tested 3 weeks after the injection.

### Immunohistochemistry and immunofluorescence

Mice brain tissues were fixed with 4% paraformaldehyde, while the cells were fixed with methanol. Then the brain was dehydrated with 30% sucrose, embedded with OCT compound (SAKURA), and sectioned to 30 μm in freezing microtome before treating with antigen retrieval solution (P0090, Beyotime). For BrdU staining, sections were immersed in 2 N HCl at 37 ℃ for 30 min, followed by 0.1 M borate buffer, pH 8.5, for 10 min at room temperature. The following steps are basically the same. Both the sections and cells were penetrated in 0.3% Triton X-100 in PBS for 30 min at room temperature, blocked in PBS containing 10% bovine serum albumin for 1 h at room temperature, incubated with primary antibodies at 4 ℃ overnight and secondary antibodies at room temperature for 2 h. For immunofluorescence, the cells spread on the well plate were fixed with 4% paraformaldehyde and incubated with primary antibody. The antibodies used were as follows: anti-Mettl1 (1:100; Proteintech; 14994-1-AP), anti-Wdr4 (1:200; Invitrogen; MA5-37987), anti-Sptbn2 (1:50; Santa Cruz; sc-515,737), anti-GFAP (1:200; Abcam; ab4674), anti-Nestin (1:250; Proteintech; 66259-1-Ig), anti-beta III Tubulin (1:200; Abcam; ab78078), anti-NeuN (1:200; Abcam; ab104224), anti-BruU (1:200; CST; 5292 S), anti-Doublecortin (1:400; CST; 4604 S).

### 5-Bromo-2′-deoxyuridine and 5-ethynyl-2′-deoxyuridine labeling

Prior to execution of the animals, BrdU (Sigma) was injected intraperitoneally at 100 mg/kg per day for 1 week. For EdU labeling, cells were treated with kFluor488-EdU method cell proliferation assay kit (KGA331, KeyGEN BioTECH) according to the manufacturer’s protocols.

### Western blotting

The different treated NSCs were collected into Eppendorf tubes, respectively. Then the cells were resuspended by adding RIPA Lysis Buffer (KGP702, KeyGEN BioTECH) containing protease inhibitors before ultrasonic fragmentation. The supernatant was extracted by centrifugation. Afterwards, the protein concentration of each sample was leveled using the BCA Protein Assay Kit (23,227, Thermo Fisher Scientific), and the loading buffer (NP0007, Invitrogen) was added and boiled before placing in -80 °C refrigerator. 40–100 µg protein samples were loaded on the SDS-PAGE gel for electrophoresis, and then transferred the separated protein onto PVDF membranes (ISEQ00010, Millipore). The membranes were blocked with 5% bovine serum albumin for 60 min and incubated overnight at 4℃ with the corresponding primary antibody, followed by 2 h of incubation with HRP-conjugated secondary antibody. After covering the membrane with Immobilon Western HRP Substrate (WBKLS0500, Millipore), the protein bands were detected using a dedicated chemiluminescent imaging system (Syngene). The following antibodies were used in Western blotting: anti-Mettl1 (1:1000; Proteintech; 14994-1-AP), anti-Wdr4 (1:1000; Invitrogen; MA5-37987), anti-Sptbn2 (1:500; Santa Cruz; sc-515,737), anti-β-actin (1:2000; CST; 4970 S), anti-rabbit HRP-linked IgG (1:2000; CST; 7074 S), anti-mouse HRP-linked IgG (1:2000; CST; 7076 S).

### m^7^G dot blot assay

mRNA was purified from the total RNA using the mRNA purification kit (Invitrogen, 61,006), followed by uniformly spotting the same quality on the nylon membrane (Invitrogen, AM10104). After cross-linking with 254 nm UV, the membrane was blocked with 5% nonfat milk in TBST and then incubated with m^7^G specific antibody (1:1000; MBL; RN017M) at 4℃ overnight.

### RNA stability assay

Actinomycin D (Sigma-Aldrich) at 5 µg/ml was added to NSCs culture. Cells were collected after 0, 3 or 6 h of incubation, followed by isolating the RNAs for QRT-PCR.

### Quantitative real-time RT-PCR

Total RNA was isolated from the cells using TRIzol reagent (Invitrogen), followed by reverse transcription using RT reagent kit with gDNA eraser (Takara, RR047A) and real-time PCR analysis using TB Green Premix Ex Taq II (Takara, RR820A). The primer sequences were as follows: Mettl1 forward, 5′-TCATCAGCCCCACACTTCTG-3′; Mettl1 reverse, 5′-CAACGGGGTCTTCACTTAGC-3′; Wdr4 forward, 5′-TCTCCAAGTCTGGCCGCTAT-3′; Wdr4 reverse, 5′-CGCACCACCATCCTGACACT-3′; Sptbn2 forward, 5′-TGACCCTTGGGCTAGTGTGGAC-3′; Sptbn2 reverse, 5′-GGCATCCTTGGCTGACTTCTTCTC-3′; GAPDH forward, 5′-GCGAGATCCCGCTAACATCA-3′; GAPDH reverse, 5′-CTCGTGGTTCACACCCATCA-3′.

### Polysome profiling

The translation efficiency of mRNA was detected by polysome profiling. In brief, the cells were incubated with 100ug/ml of actinomycin for 15 min before collection, followed by adding lysis buffer. Subsequently, the centrifuged lysate was added to a prepared 10–45% sucrose density gradient and centrifuged at 36 000 rpm for 3 h, followed by separation with the gradient density separator. RNA was extracted from each fraction and the relative expression of Sptbn2 mRNA on the polysome fraction was detected by QRT-PCR.

### m^7^G-MeRIP-seq and data process

The m^7^G-IP-Seq service was provided by CloudSeq Inc. (Shanghai, China). Total RNA was subjected to immunoprecipitation with the GenSeq® m^7^G-IP Kit (GenSeq Inc.) by following the manufacturer’s instructions. Briefly, RNA was decapping with Tobacco Decapping Enzyme, and then randomly fragmented to about 200 nt by RNA Fragmentation Reagents. Protein A/G beads were coupled to the m^7^G antibody by rotating at room temperature for 1 h. The RNA fragments were incubated with the bead-linked antibodies and rotated at 4℃ for 4 h. The RNA/antibody complexes are then digested with Proteinase K and the eluted RNA is purified by phenol:chloroform extraction. RNA libraries for IP and input samples were then constructed with NEBNext® Ultra II Directional RNA Library Prep Kit (New England Biolabs, Inc.) by following the manufacturer’s instructions. Libraries were qualified using Agilent 2100 bioanalyzer and then sequenced in a NovaSeq platform (Illumina).

Briefly, paired-end reads were harvested from Illumina novaseq 6000 sequencer, and were quality controlled by Q30. After 3’ adaptor-trimming and low quality reads removing by cutadapt software (v1.9.3). First, clean reads of all libraries were aligned to the reference genome (UCSC RN5) by Hisat2 software (v2.0.4). Methylated sites on RNAs (peaks) were identified by MACS software. Differentially methylated sites were identified by diffReps. These peaks identified by both software overlapping with exons of mRNA were figured out and chosen by home-made scripts. GO and Pathway enrichment analysis were performed by the differentially methylated protein coding genes.

### Morris water maze

The spatial learning and memory ability of mice were assessed using the Morris water maze. Briefly, mice were placed in a pool (120 cm in diameter) to find a hidden platform (10 cm in diameter and 1 cm underwater) within 60 s. After the mice stayed on the platform for 2 s, stopped timing and recorded as escape latency. The spatial learning task consisted of 5 days training with 4 trials (60 s in duration) per day to find the hidden platform. Different patterns on the pool walls were used to ensure that mice could use visual-spatial memory to find the hidden platforms. On the sixth day, we removed the platform, followed by observing and recording the mice’s movement trace, the number of times they crossed the platform and the time they stayed in the quadrant of the platform. All of the behavioral parameters of the mice were tracked, recorded, and analyzed using Ethovision XT 14.0 software (Noldus).

### Statistical analysis

All data were analyzed using statistical software SPSS 16.0 and GraphPad Prism 8.0. The unpaired student’s t-test was used to determine the difference between the two groups. One-way ANOVA analysis and Bonferroni multiple comparison test were used to determine the difference between the multiple groups. Escape latencies during spatial learning in the Morris water maze were analyzed via two-way ANOVA. All experiments were repeated three times, and *P* < 0.05 was considered significant.

## Results

### m^7^G methyltransferases Mettl1/Wdr4 show differential expressions during neurogenesis

**Fig. 1:**
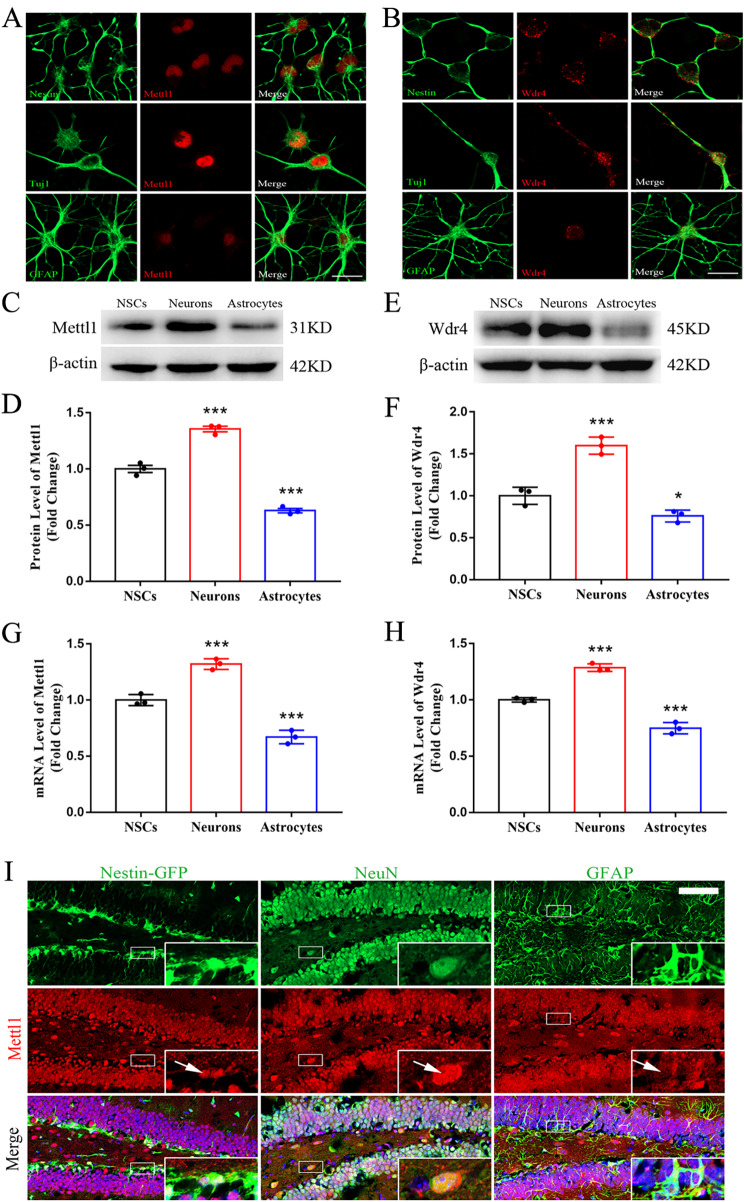
**Differential expression of Mettl1 and Wdr4 during neurogenesis**. (**A, B**) Immunofluorescence staining for Mettl1 and Wdr4 in Nestin + NSCs, Tuj1 + neurons and GFAP + astrocytes. Scale bar, 150 μm. (**C, D**) Western blotting and quantification for Mettl1 in NSCs, neurons and astrocytes. n = 3, ***P < 0.001 compared with NSCs. (**E, F**) Western blot and quantification for Wdr4 in NSCs, neurons and astrocytes. n = 3, ***P < 0.001, *P < 0.05 compared with NSCs. (**G, H**) The expressions of Mettl1 and Wdr4 mRNA in NSCs, neurons and astrocytes. n = 3, ***P < 0.001 compared with NSCs. (**I**) Immunofluorescence staining for Mettl1 costaining with Nestin, NeuN and GFAP in dentate gyrus of hippocampus. Scale bar, 200 μm. Data are represented as the mean ± SEM. NSCs, neural stem cells; n represents number of independent experiments

To unveil the potential function of m^7^G methyltransferases in NSCs, we first assessed the expression of Mettl1/Wdr4 in NSCs, neurons and astrocytes derived from NSCs. Immunostaining revealed high Mettl1/Wdr4 protein expression in the nuclei of Nestin^+^ and Tuj1^+^ cells and low expression in GFAP^+^ cells (Fig. 1A, B). Further, Western blotting and RT-qPCR analyses confirmed that the expression of Mettl1/Wdr4 in neurons was remarkably increased compared to NSCs, whereas strikingly decreased in astrocytes and the results was in agreement with immunostaining data (Fig. 1C-H), implying that m^7^G methyltransferases are closely associated with neurogenesis. To further validate our in vitro findings, the m^7^G methyltransferase was co-stained with NSCs, neurons and astrocytes in mouse hippocampus. Interestingly, we observed that Mettl1 immunostaining was more intense in the nucleus of NSCs and neurons (Fig. 1I). These findings imply a critical role of m^7^G methyltransferases in regulating neurogenesis.

### Mettl1 facilitates neuronal differentiation of NSCs

**Fig. 2:**
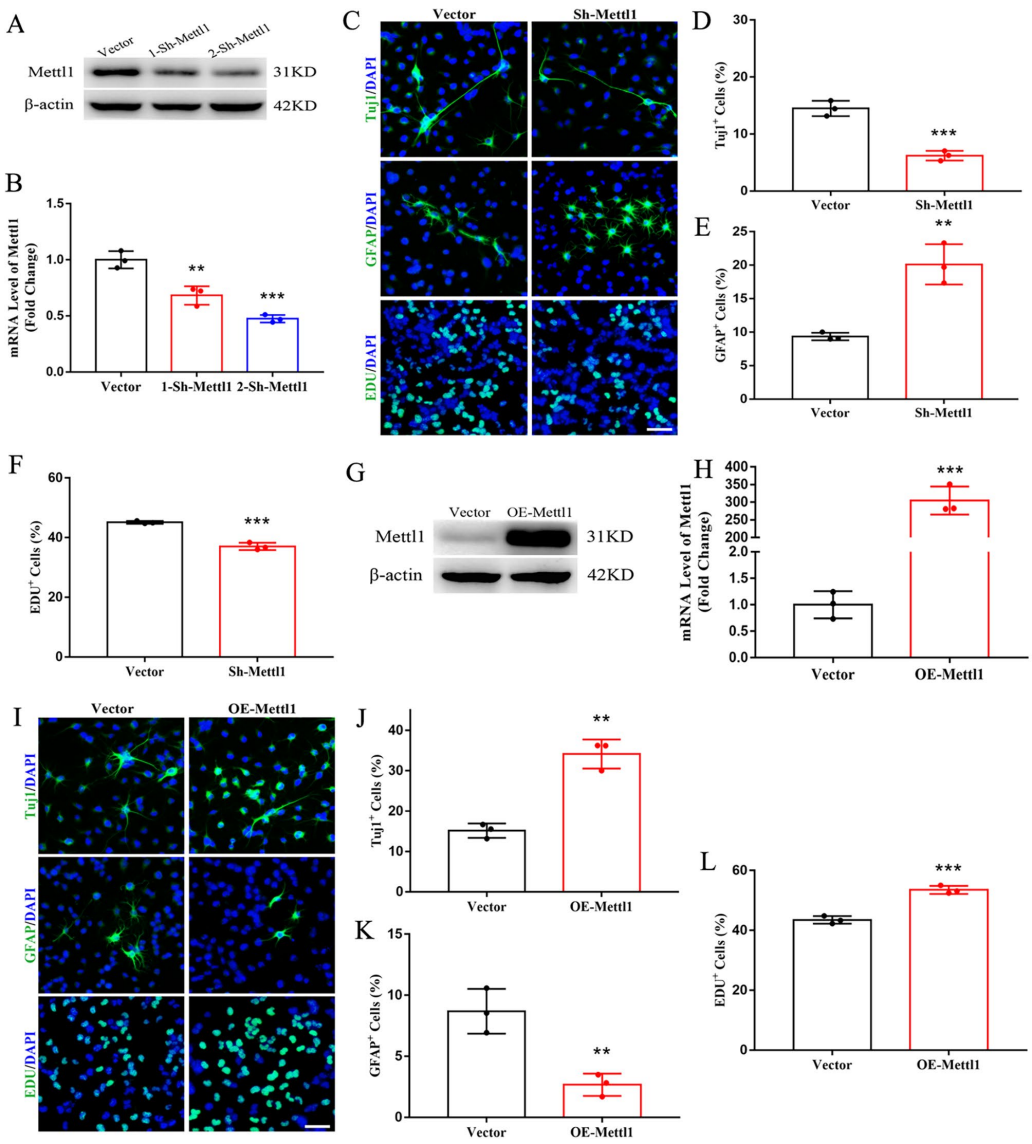
**Mettl1 promotes neuronal differentiation of NSCs.**. (**A,B**) Western blotting and RT-qPCR of Mettl1 expression in NSCs treated with Mettl1 shRNAs. n = 3, ***P < 0.001, **P < 0.01 compared with control. (**C-F**) Immunofluorescence staining and quantification for Tuj1, GFAP and EdU on NSCs treated with control or Mettl1 shRNA. Scale bar, 200 μm. n = 3, ***P < 0.001, **P < 0.01 compared with control. (**G,H**) Western blotting and RT-qPCR of Mettl overexpression in NSCs. n = 3, ***P < 0.001 compared with control. (**I-L**) Immunofluorescence staining and quantification for Tuj1, GFAP and EdU on NSCs treated with control or Mettl1 overexpression. Scale bar, 200 μm. n = 3, ***P < 0.001, **P < 0.01 compared with control. Data are represented as the mean ± SEM. NSCs, neural stem cells; Sh-Mettl1, shRNA Mettl1; OE-Mettl1, overexpressing Mettl1; n represents number of independent experiments

To investigate the biological significance of m^7^G methyltransferase in NSCs, we designed shRNA to knock-down Mettl1 (Fig. 2A, B). Neuronal differentiation and NSCs proliferation were dramatically inhibited upon Mettl1 depletion. Meanwhile, glial cell differentiation was strongly elicited (Fig. 2C-F). In contrast, Mettl1 overexpression enhanced neuronal differentiation and NSCs proliferation (Fig. 2G-L). These results indicate an induction of neurogenesis and suppression of astrogliogenesis by m^7^G methyltransferase.

### Mettl1 mediated m^7^G methylation in neurogenesis

**Fig. 3:**
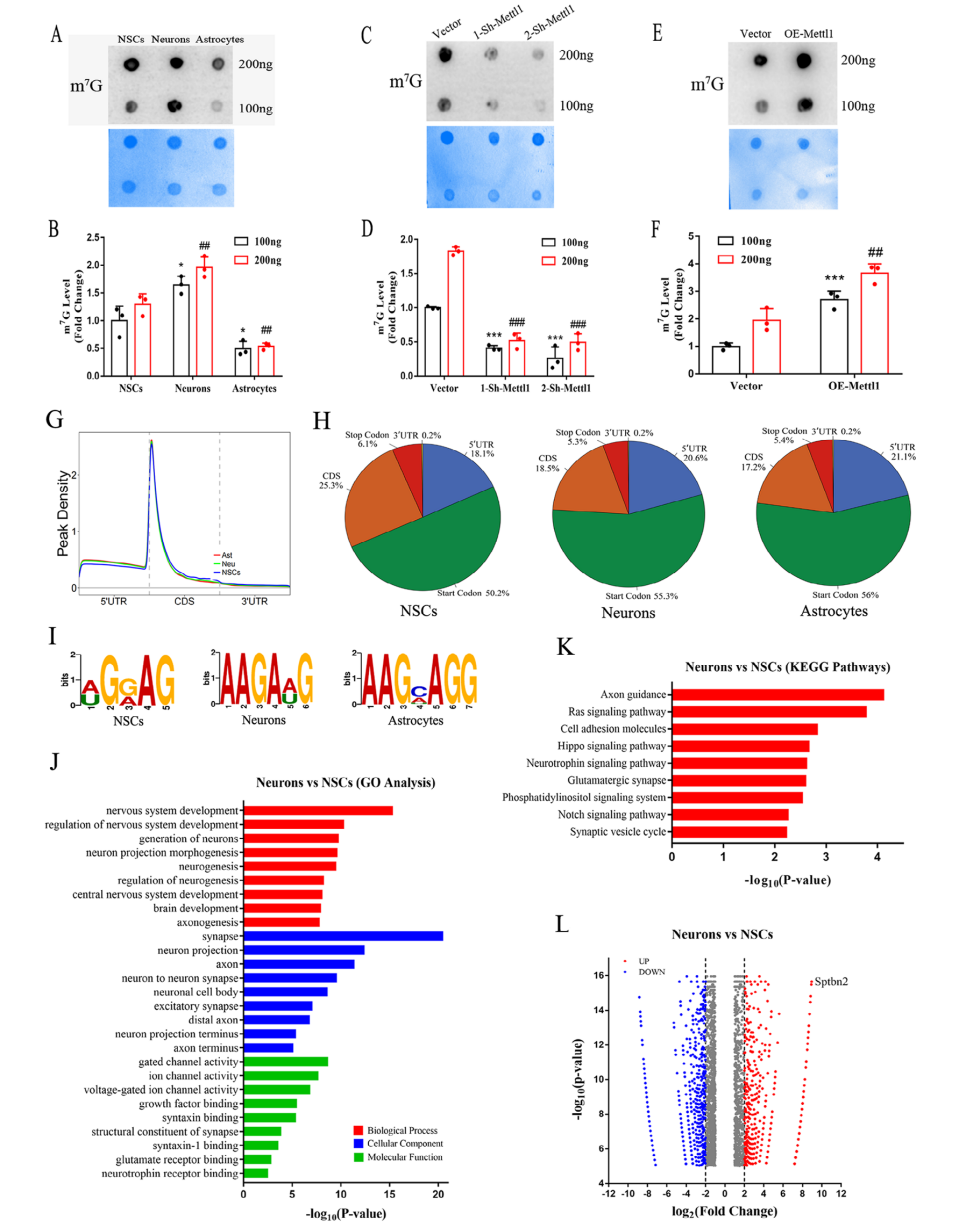
**Mettl1 modulates the transcriptome landscape of internal m**^**7**^**G-modified mRNA during neurogenesis**. (**A, B**) RNA dot blot analysis of m^7^G levels in NSCs, astrocytes and neurons. n = 3, *P < 0.05 compared with NSCs (100 ng); ##*P* < 0.01 compared with NSCs (200 ng). (**C, D**) RNA dot blot analysis of m^7^G levels in NSCs treated with Mettl1 shRNAs. n = 3, ***P < 0.001 compared with NSCs (100 ng); ###*P* < 0.001 compared with NSCs (200 ng). (**E, F**) RNA dot blot analysis of m^7^G levels in NSCs treated with Mettl1 overexpression. n = 3, ***P < 0.001 compared with NSCs (100 ng); ##*P* < 0.01 compared with NSCs (200 ng). (**G**) Distribution of internal m^7^G across NSCs, neurons and astrocytes mRNA segments. (**H**) Pie charts presenting the fraction of m^7^G peaks in NSCs, neurons and astrocytes. (**I**) Motif analysis of internal mRNA m^7^G in NSCs, neurons and astrocytes mRNAs (**J, K**) Bar plot chart showing the significant GO terms and KEGG analysis for NSCs and differentiated neurons mRNAs containing internal m^7^G. (**L**) Volcano plot of significantly altered internal mRNA m^7^G peaks in NSCs compared to neurons. NSCs, neural stem cells; Neu, neurons; Ast, astrocytes. Data are represented as the mean ± SEM. NSCs, neural stem cells; Sh-Mettl1, shRNA Mettl1; OE-Mettl1, overexpressing Mettl1; n represents number of independent experiments

Considering that Mettl1 is a stable m^7^G methyltransferase, we next investigated whether Mettl1 is involved in the induction of m^7^G formation upon neurogenesis and found that a obvious increase of the m^7^G levels in NSCs-derived neurons and significant decrease in NSCs-derived astrocytes compared to NSCs (Fig. 3A, B), suggesting distinct role of m^7^G in determining cell fate. Moreover, Mettl3 depletion significantly reduced m^7^G level compared with the control (Fig. 3C, D). Conversely, overexpressing Mettl1 amazingly increased m^7^G level (Fig. 3E, F), revealing Mettl1-dependent m^7^G methylation in NSCs. Overall, our results highlight a close correlation between Mettl1 and m^7^G level changes during neurogenesis, indicating a potential role of Mettl1 in modulating m^7^G formation.

### Identification of m^7^G targets during neurogenesis

The existence of internal m^7^G along mRNA reveals a potential function of the modification [7]. To elucidate the mechanisms underlying internal m^7^G mRNA modification in the regulating neurogenesis, we performed m^7^G mRNA meRIP-seq on 5′cap digested mRNAs from NSCs, neurons and astrocytes to analyse the dynamic profiles of internal mRNA m^7^G methylome. We observed that internal m^7^G peaks are enriched at 5′UTR in close proximity to translation initiation site (TIS) (Fig. 3G). To further validate the preferential landscape of m^7^G on transcripts, we generated the distribution of internal m^7^G reads and consistently found that m^7^G were mainly located within the start codon and then 5′UTR (Fig. 3H). Intriguingly, motif analysis of internal mRNA m^7^G from three types of cells displayed a similar preference to AG-rich regions (Fig. 3I). Altogether, these results strongly support successful identification of the specific m^7^G sites in neurogenesis.

Next, we sought to identify direct m^7^G targets in neurogenesis via m^7^G mRNA MeRIP-Seq in NSCs compared with neurons. Gene ontology (GO) analysis revealed that m^7^G-modified mRNAs were enriched in neurogenesis, such as nervous system development, neuron differentiation and brain development (Fig. 3J). KEGG analysis revealed that differentially expressed genes (DEGs) were enriched in the axon guidance, ras signaling and hippo signaling pathways (Fig. 3K). In addition, GO analysis the m^7^G-tagged internal mRNA showed an enrichment of genes related to negative regulation of cellular process and biological process (Additional file 1: Fig. S1A). KEGG enrichment analysis of the RNA-seq data revealed m^7^G-tagged internal mRNA to be significantly associated with the MAPK, rap1 and mTOR signaling pathways in NSCs compared with astrocytes (Additional file 1: Fig. S1B). Of all m^7^G peaks, 1224 were upregulated, while 1724 were downregulated in neurons compared with NSCs (Fig. 3L). Additionally, 878 upregulated and 2036 downregulated peaks were found in astrocytes compared with NSCs (Additional file 1: Fig. S1C, 1D). Collectively, the data reveal the dynamic and diverse m^7^G changes in neurogenesis.

### Internal m^7^G promotes Sptbn2 stability and translation

**Fig. 4:**
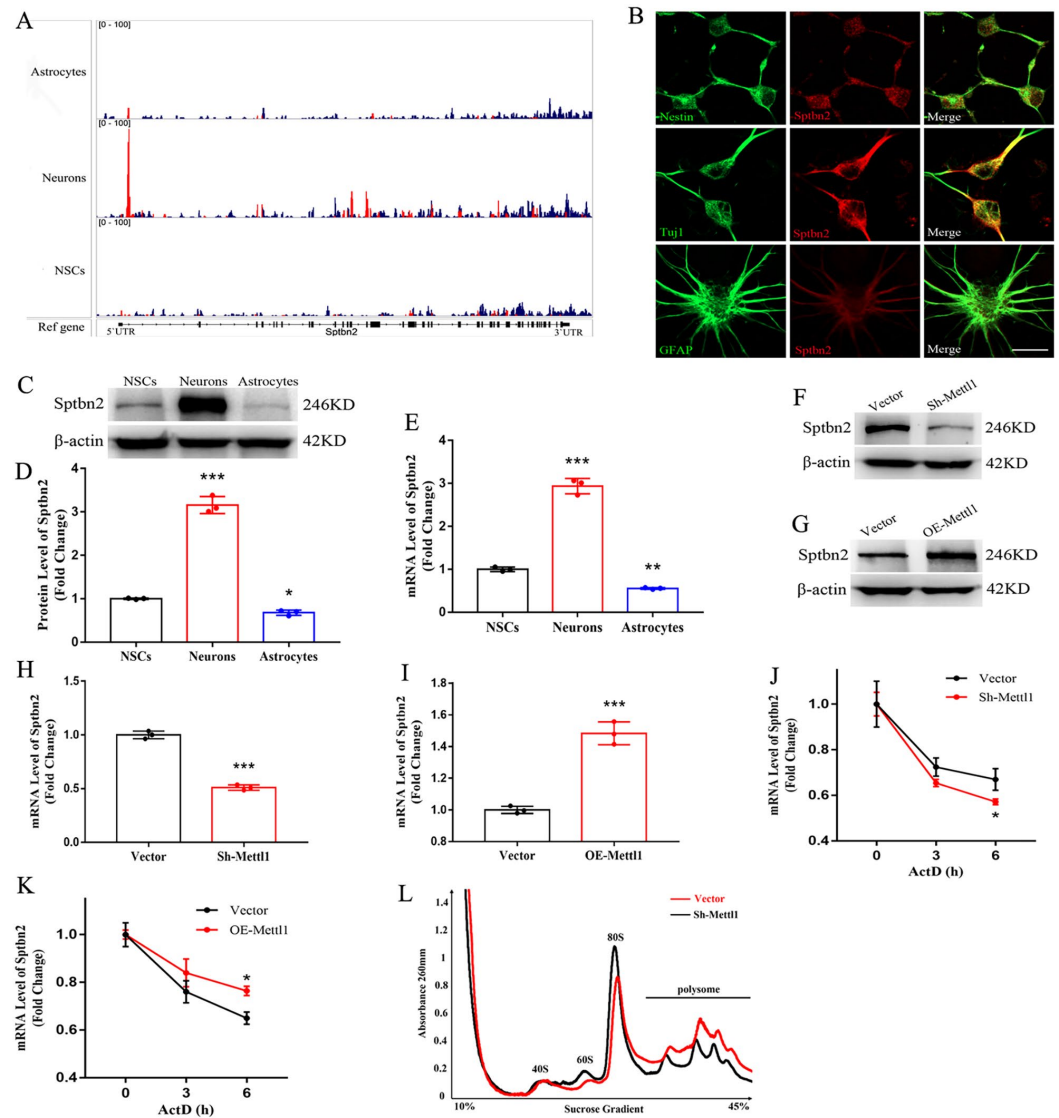
**Internal mRNA m**^**7**^**G promotes Sptbn2 stability and translation**. (**A**) Integrative Genomics Viewer (IGV) tracks displaying m^7^G reads from NSCs, neurons and astrocytes. (**B-E**) Immunofluorescence staining, Western blotting and RT-qPCR of Sptbn2 expression in NSCs, neurons and astrocytes. Scale bar, 150 μm, n = 3, ***P < 0.001, **P < 0.01, *P < 0.05 compared with NSCs. (**F-I**) The expression of Sptbn2 protein and mRNA in NSCs transfected with the depletion or overexpression of Mettl1. n = 3, ***P < 0.001 compared with control. (**J, K**) Sptbn2 mRNA stability was determined by qRT-PCR in control and NSCs transfected with the depletion or overexpression of Mettl1 using the samples treated with ActD at the indicated times. n = 3, *P < 0.05 compared with control. (**L**) Polysome profiling of NSCs with or without Mettl1 knockdown. Data are represented as the mean ± SEM. NSCs, neural stem cells; Sh-Mettl1, shRNA Mettl1; OE-Mettl1, overexpressing Mettl1; ActD, actinomycin D; n represents number of independent experiments

Comparison of m^7^G profiles in neurons and NSCs verified many genes related to neurogenesis as m^7^G targets, including Sptbn2 (Fig. 3L). Integrative Genomics Viewer (IGV) demonstrated that neurogenesis-associated Sptbn2 gene transcript displayed a strong enrichment of internal m^7^G in 5′UTR and CDS in neurons (Fig. 4A), suggesting the crucial role of internal m^7^G modification of Sptbn2 in regulating neurogenesis.

To assess whether internal m^7^G affects Sptbn2 expression, we first determined Sptbn2 expression in 3 types of cells. Interestingly, the data from immunofluorescence, western bloting and RT-qPCR showed that Sptbn2 was heavily expressed in neurons and weakly expressed in astrocytes compared with NSCs (Fig. 4B-E), indicating that Sptbn2 expression was strongly associated with internal m^7^G in neuronal genesis. To further substantiate the relation, we knocked down or overexpressed Mettl1 to test Sptbn2 expression in NSCs and found that Mettl1 depletion dramatically reduced the levels of Sptbn2 protein and mRNA, while the levels of Sptbn2 protein and mRNA were significantly enhanced by forced expression of Mettl1 in NSCs (Fig. 4F-I), implying Sptbn2 a potential target of Mettl1-mediated m^7^G modification. Taken together, our results suggest that Mettl1-dependent m^7^G promotes Sptbn2 expression in neurogenesis.

We then determined whether internal m^7^G methylation regulates the stability of Sptbn2 mRNA, we performed RNA decay rates via actinomycin D decay and found that Mettl1 depletion shortened half-life of Sptbn2 mRNA (Fig. 4J). In contrast, forced expression of Mettl1 prolonged the Sptbn2 mRNA half-life (Fig. 4K). Altogether, our results indicate that Mettl1-mediated internal m^7^G modification facilitates Sptbn2 expression via enhancing Sptbn2 mRNA stability. Subsequently, we further elucidated whether internal m^7^G affect mRNA translation via polysome profiling and found that Mettl1 depletion induced a decrease of the polyribosome peak (Fig. 4L), suggesting that Mettl1-mediated m^7^G mRNA modification may regulate the Sptbn2 mRNA translation in neurogenesis.

### Sptbn2 is required for Mettl1-induced neurogenesis

**Fig. 5:**
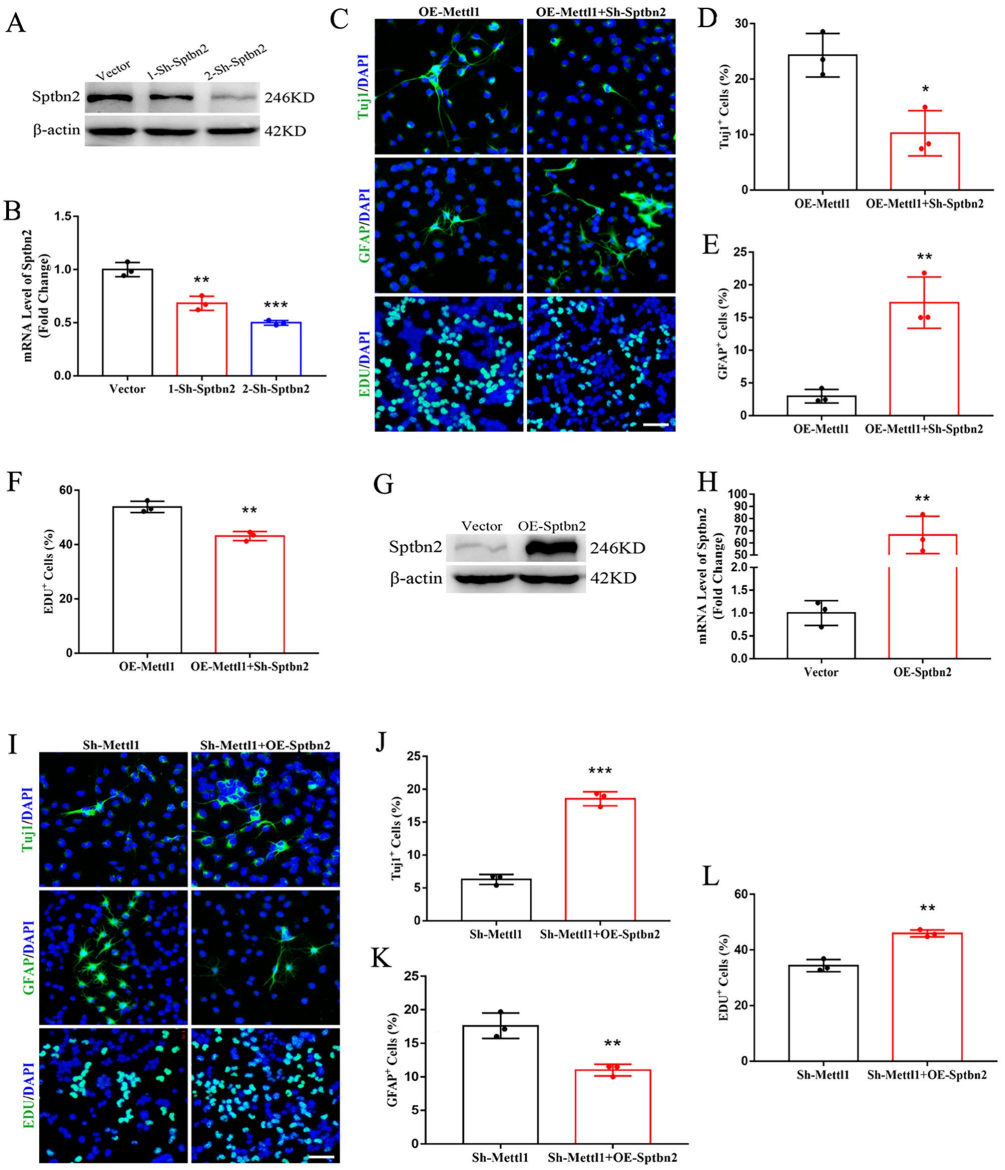
**Sptbn2 mediates Mettl1-induced neurogenesis**. (**A, B**) Western blotting and RT-qPCR of Sptbn2 to validate the knockdown efficiency in NSCs. **P < 0.01, ***P < 0.001 compared with Vector. (**C-F**) Immunofluorescence staining and quantification for Tuj1, GFAP, and EdU on NSCs treated with overexpressing Mettl1 or shRNA Sptbn2. Scale bar, 200 μm. n = 3, *P < 0.05, **P < 0.01 compared with Mettl1 overexpression. (**G, H**) Western blotting and RT-qPCR of Sptbn2 to confirm the overexpression efficiency in NSCs. **P < 0.01 compared with Vector. (**I-L**) Immunofluorescence staining and quantification for Tuj1, GFAP, and EdU on NSCs treated with shRNA Mettl1 or overexpressing Sptbn2. Scale bar, 200 μm. n = 3, **P < 0.01, ***P < 0.001 compared with shRNA Mettl1. Data are represented as the mean ± SEM. Sh-Mettl1, shRNA Mettl1; Sh-Sptbn2, shRNA Sptbn2; OE-Mettl1, overexpressing Mettl1; OE-Sptbn2, overexpressing Sptbn2; n represents number of independent experiments

Considering the positive correlation between Mettl1 and Sptbn2, we further explore the biological significance of Sptbn2 in the NSCs differentiation defects induced by the overexpression or depletion of Mettl1. We performed rescue experiments and found that Sptbn2 knockdown reversed the increased neurogenesis caused by forced expression of Mettl1 (Fig. 5A-F). Conversely, Sptbn2 overexpression restored the differentiation defect of Mettl1-deficient NSCs (Fig. 5G-L). Overall, these results strongly demonstrated that Mettl1-mediated internal m^7^G modulates neurogenesis through impacting Sptbn2 expression.

### Mettl1 knockdown elicits defective neurogenesis and cognitive impairment

**Fig. 6:**
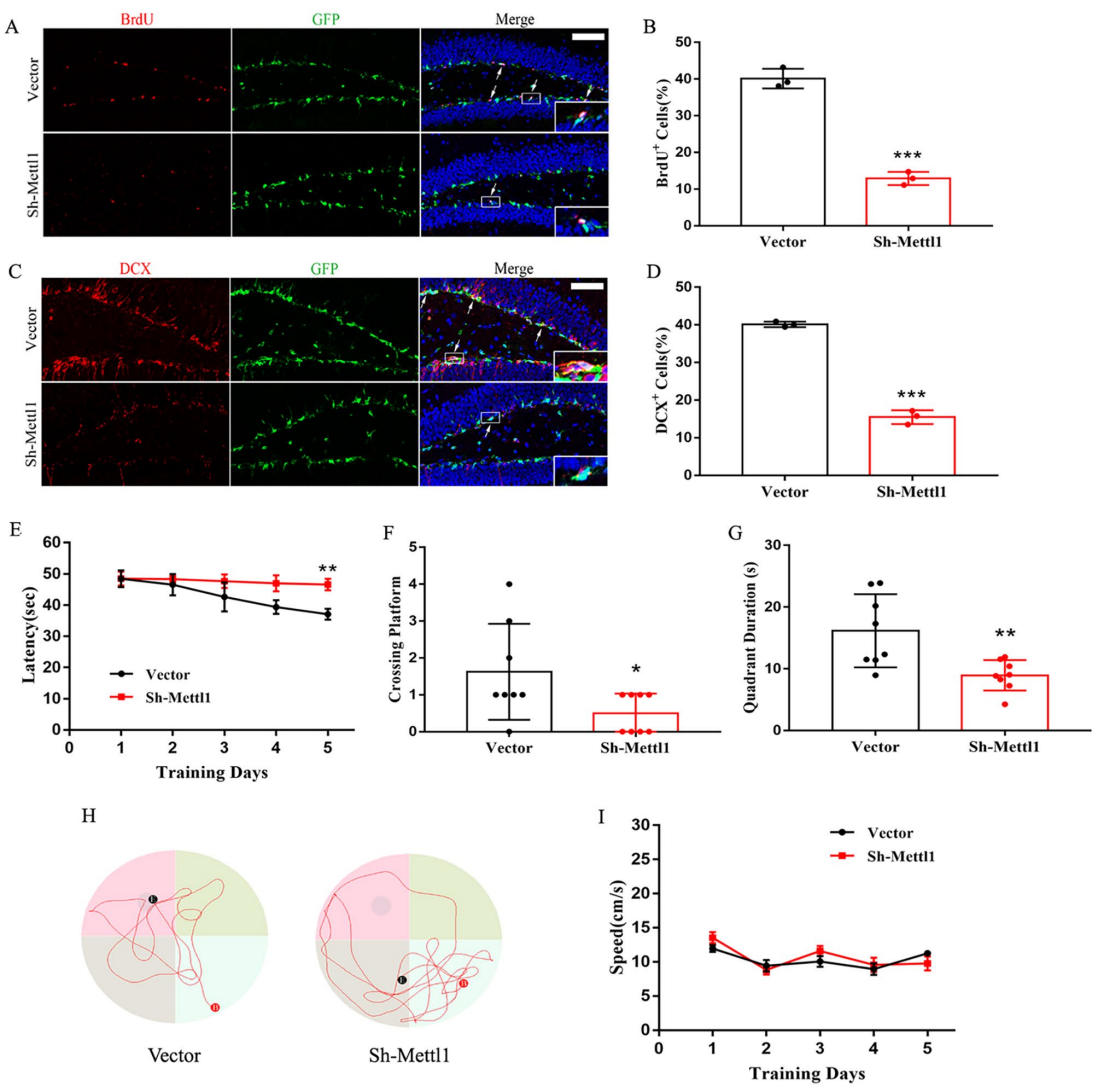
**Mettl1 knockdown elicits reduced neurogenesis and cognitive impairment in mice**. **(A-D**) Representative fluorescence images and quantification for BrdU and DCX after Mettl1 shRNA retrovirus infection. Scale bar, 250 μm. ***P < 0.001 compared with vector (control). (**E**) The escape latency in the navigation test. **P < 0.01 compared with vector (control). (**F**) The number of target crossings where the platform had been located in the probe test. *P < 0.05 compared with vector (control). (**G**) Quadrant time (%) in the probe test. **P < 0.01 compared with vector (control). (**H**) Representative swimming paths in each quadrant during the probe trial. (**I**) The average swimming speed between the two groups. n = 8 mice per group. Data are represented as the mean ± SEM. Sh-Mettl1, shRNA Mettl1

To further substantiate our in vitro findings, we then determined whether Mettl1 affected hippocampal neurogenesis in vivo and delivered retroviruses expressing Mettl1 shRNA to the dentate gyrus of hippocampus via bilateral stereotactic injection. We observed that Mettl1 depletion dramatically decreased neurogenesis (BrdU^+^/DCX^+^ cells) (Fig. 6A-D), indicating the involvment of Mettl1 in adult hippocampal neurogenesis.

Hippocampal neurogenesis is closely associated with cognitive function, we hypothesized that decreased neurogenesis in Mettl1-deficency mice might induce cognitive impairment. To address this possibility, we employed Morris water maze to assess spatial learning and memory of the mice and found that Mettl1-deficency mice showed a markedly cognitive decline during the last one session in the acquisition trial (Fig. 6E), implying an impaired learning ability. In the probe trial, Mettl1-deficency mice displayed fewer the number of crossing over the platform compared to the control mice, spending less time in target quadrant despite being similar in swimming velocity (Fig. 6F-I), indicating defects in spatial memory.

### Overexpressing Mettl1 rescues the defective neurogenesis and cognitive impairment in APP/PS1 mice

**Fig. 7:**
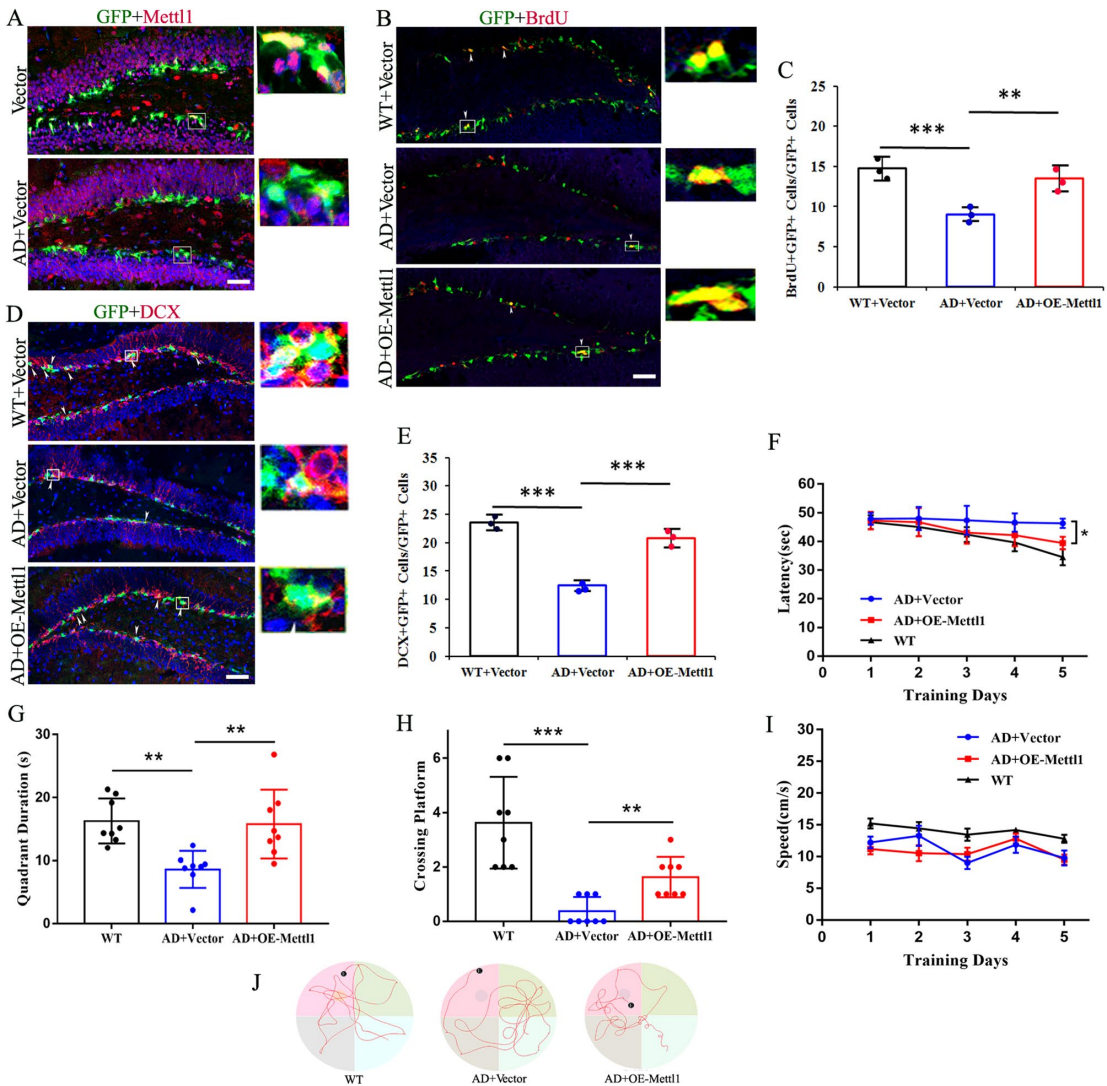
**Mettl1 overexpression rescues the decreased neurogenesis and cognitive deficit in APP/PS1 mice**. (**A**) Representative fluorescence images for Mettl1 in the NSCs of hippocampus. Scale bar, 200 μm. (**B-E**) Representative fluorescence images and quantification for BrdU and DCX after retrovirus-mediated Mettl1 overexpression. Scale bar, 250 μm. ***P < 0.001, **P < 0.01. (**F**) The escape latency in the navigation test. *P < 0.05 compared with AD + vector. (**G**) Quadrant time (%) in the probe test. **P < 0.01. (**H**) The number of target crossings where the platform had been located in the probe test. ***P < 0.001, **P < 0.01. (**I**) The average swimming speed among the groups. (**J**) Representative swimming paths in each quadrant during the probe trial. n = 8 mice per group. Data are represented as the mean ± SEM. AD, Alzheimer’s disease (APP/PS1 mice); OE-Mettl1, overexpressing Mettl1

As Mettl1-deficency mice exhibited decreased neurogenesis and cognitive impairment, we next investigated whether forced expression of Mettl1 improved hippocampal neurogenesis and cognitive function in APP/PS1 mice. We first evaluated the level of Mettl1 expression in hippocampal NSCs and found that weak expressions were observed in the NSCs of APP/PS1 mice compared with control (Fig. 7A). Furthermore, Mettl1 overexpression significantly enhanced hippocampal neurogenesis (BrdU+/DCX + cells) of APP/PS1 mice (Fig. 7B-E), demonstrating crucial role of Mettl1 in hippocampal neurogenesis. Additionally, forced expression of Mettl1 remarkably ameliorated cognitive impairment in APP/PS1 mice (Fig. 7F-J). Collectively, our data indicate Mettl1 as a novel anti-Alzheimer disease target.

## Discussion

The mRNA cap N7-methylguanosine (m^7^G) installed by Mettl1/Wdr4 complex is a positively charged and evolutionarily conserved modification during transcription initiation in mammals, which stabilizes transcripts [12, 13] and modulates mRNA export [14], translation [15], and splicing [16]. m^7^G was newly revealed to occur in internal mRNA, mainly in 5′UTR, coding sequence (CDS) and 3′UTR [5]. Accumulative evidence demonstrates the pivotal roles of internal m^7^G in pathological and physiological processes of various cell lines and brain issues including HeLa, HepG2, mESC, MEF and 293T cells [5, 7]. Internal mRNA m^7^G displayed relatively increased enrichment in the CDS and 3′UTR regions versus a decrease in 5′UTR regions upon heat shock and oxidative stress [7]. Furthermore, Mettl1-mediated m^7^G within mRNA promotes mRNA translation efficiency, indicating that internal m^7^G can serve as a novel epitranscriptomic regulator for translation and stress response. However, the role of internal mRNA m^7^G methylome in hippocampal neurogenesis remains unknown. Here, we uncovered the existence and distribution of m^7^G within mRNA in NSCs, neurons and astrocytes, and address this notion that internal m^7^G methylation of Sptbn2 enhanced its stability and translation, which contributed to Mettl1-induced neurogenesis. However, the function and precise mechanisms of m^7^G methylation within tRNA and rRNA in adult hippocampal neurogenesis need to be further investigated.

The Sptbn2 gene encoding β-III spectrin, a cytoskeletal protein, exists widely throughout the brain and is related to intracellular transport, Golgi apparatus and cytoplasmic vesicles [17, 18]. Sptbn2 deficiency in the brain of the human and mouse results in neuronal dysfunction in widespread brain regions [19, 20]. Three heterozygous mutations of Sptbn2 cause Spinocerebellar Ataxia Type 5 (SCA5) in humans, a neurodegenerative disorder leading to impaired brain development and loss of motor coordination [21], while homozygous mutations result in a more severe childhood ataxia with cognitive impairment [19, 22], indicating an crucial role of Sptbn2 in neurodevelopment and cognition. Our results reveal that Sptbn2 is strongly enriched in neurons, and weakly expressed in astrocytes compared to NSCs, suggesting that Sptbn2 is closely associated with neuronal differentiation. However, it remains unresolved how is the functional relationship of internal m^7^G modification and Sptbn2. This connection is validated by our finding that m^7^G methyltransferase Mettl1 catalyzes targeted Sptbn2 mRNA in Mettl1-induced neurogenesis. Additionally, we further identify that Mettl1-mediated internal m^7^G methylation promotes Sptbn2 expression via regulating Sptbn2 mRNA stability and translation. Strikingly, transcriptome analysis reveals the generally dynamic m^7^G modification changes at the internal mRNAs of Sptbn2 during neurogenesis. Collectively, our results raises a novel possibility that Mettl1-mediated internal m^7^G governs Sptbn2 stability and translation in Mettl1-mediated neurogenesis.

Cognitive impairment in Alzheimer’s disease (AD) is closely related to dysregulation of hippocampal neurogenesis. Increasing evidence uncovers that decreased neurogenesis contributes to cognitive decline in rodents as well as in humans [23–25], albeit the precise mechanism remains largely uncharacterized. Emerging experiments convincingly demonstrate that the efficiency of anti-AD depends on the capacity of inducing hippocampal neurogenesis [26–28], suggesting that targeting neurogenesis ameliorates cognitive dysfunction in AD. However, direct association between internal mRNA m^7^G methylation and neurogenesis remains to be established. This correlation is clearly confirmed by our findings that ectopic expression and depletion of Mettl1 mediates adult hippocampal neurogenesis in vitro and in vivo, and improves cognitive deficits of AD, indicating that Mettl1-mediated internal m^7^G dysregulation contributes to the pathophysiology of AD. Recent research discovers the biological significance of m^7^G methylation modification in AD and develops potential predictive models to assess the risk of m^7^G subtypes and the pathological outcomes of patients with AD [29]. However, more work about the direct interplay between the internal m^7^G and AD still awaits elucidation. In addition, our data revealed 1224 upregulated and 1724 downregulated m^7^G peaks in neurons compared to NSCs. Although this large footprint may cause off target effects, our results strongly suggest that overexpressing Mettl1 obviously enhanced the neurogenesis in vitro and in vivo. Altogether, current results highlight the novel function that targeting Mettl1 in promoting neurogenesis alleviates cognitive impairment of AD.

## Conclusion

In summary, we uncover a critical role of Mettl1-mediated internal m^7^G in adult hippocampal neurogenesis via enhancing Sptbn2 mRNA stability and translation. Our findings further reveals that mice with hippocampal Mettl1 deficiency displays decreased neurogenesis and cognitive impairment, whereas ectopic overexpression of Mettl1 improves neurogenesis and cognition in mouse models of AD. Our study identifies targeting Mettl1 regulation of neurogenesis as a potential therapeutic option for AD.

### Electronic supplementary material

Below is the link to the electronic supplementary material.

Supplementary Material 1

Supplementary Material 2

## Data Availability

All data will be provided upon availability and reasonable request.

## Abbreviations

m^7^G: N^7^-methylguanosine
AD: Alzheimer’s disease
NSCs: Neural stem cells
Aβ: Amyloid beta
AHN: Adult hippocampal neurogenesis
Mettl1: Methyltransferase-like 1
Wdr4: WD repeat domain 4
mESCs: Mouse embryonic stem cells
hiPSCs: Human-induced pluripotent stem cells
TIS: Translation initiation site
GO: Gene ontology
IGV: Integrative Genomics Viewer
CDS: Coding sequence
