# Supplementary material for: Mettl1-mediated internal m7G methylation of Sptbn2 mRNA elicits neurogenesis and anti-alzheimer’s disease
Source: Cell Biosci. 2023 Oct 1;13:183. doi: 10.1186/s13578-023-01131-2 (PMC10544167; doi:10.1186/s13578-023-01131-2)
Supplement: Supplementary file 1 — Supplementary Material 1 [file 13578_2023_1131_MOESM1_ESM.docx]

Figure 1C


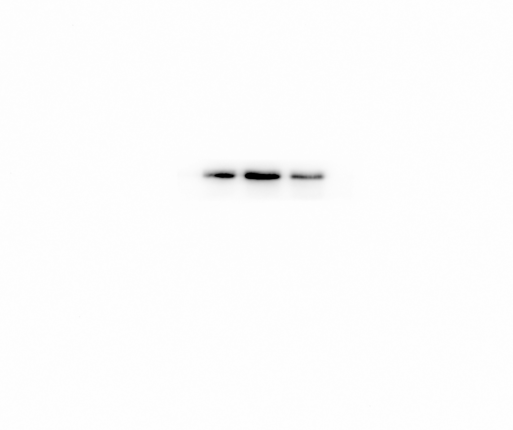

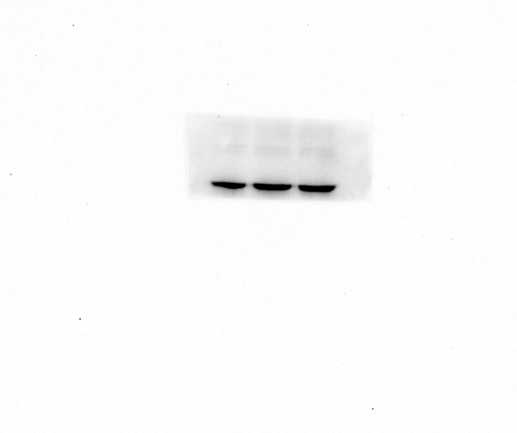


β-actin

NSCs Neurons Astrocytes

NSCs Neurons Astrocytes

Mettl1

Figure 1E


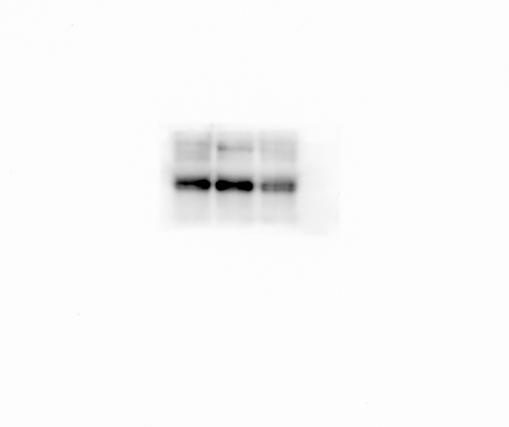

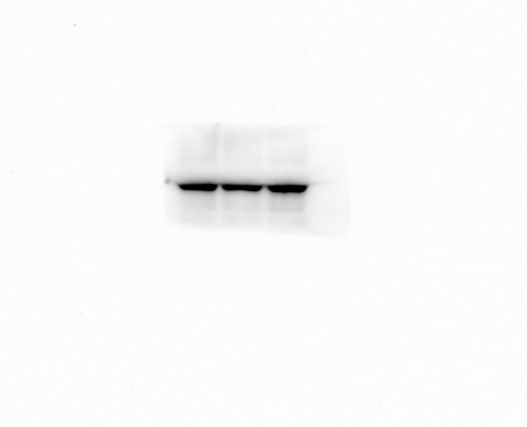


β-actin

NSCs Neurons Astrocytes

Wdr4

NSCs Neurons Astrocytes

Figure 2A


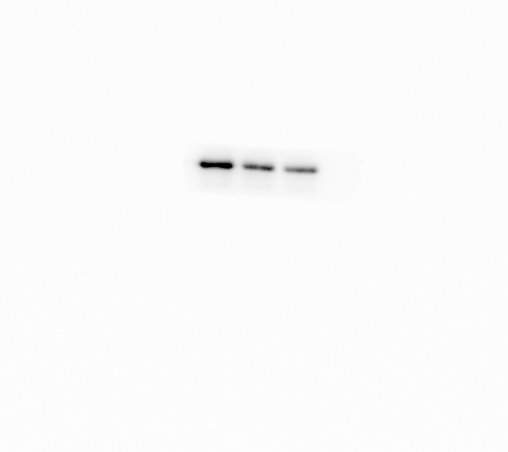

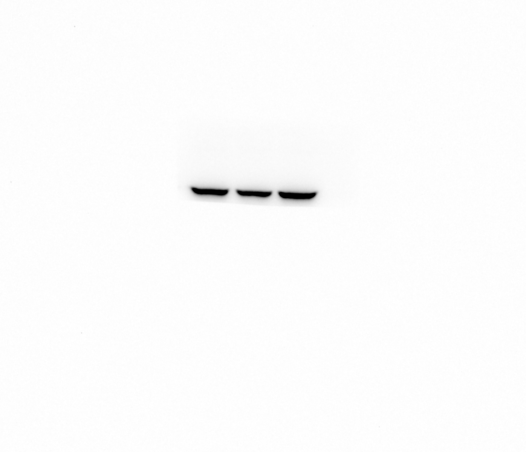


β-actin

Vector 1-sh-Mettl1 2-sh-Mettl1

Mettl1

Vector 1-sh-Mettl1 2-sh-Mettl1

Figure 2G


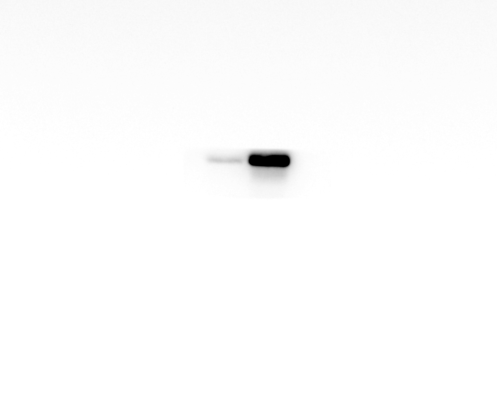

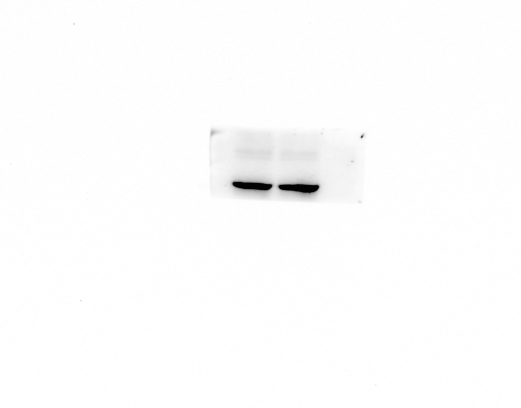


β-actin

Vector OE-Mettl1

Mettl1

Vector OE-Mettl1

Figure 5C


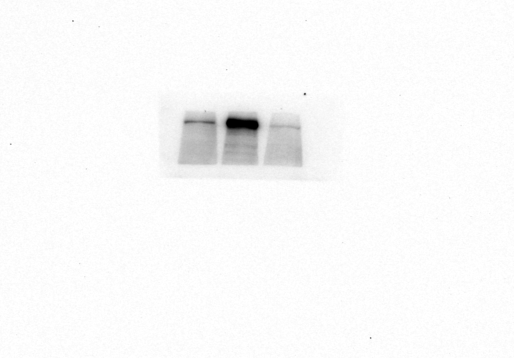

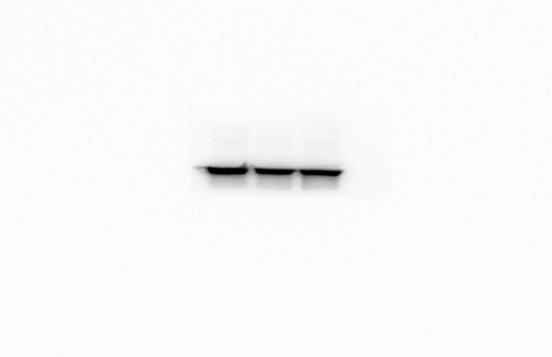


β-actin

NSCs Neurons Astrocytes

Sptbn2

NSCs Neurons Astrocytes

Figure 5F


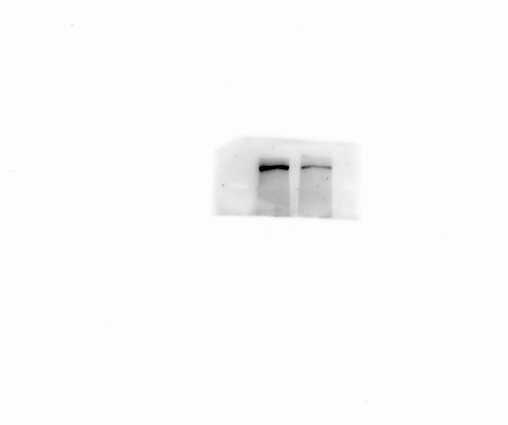

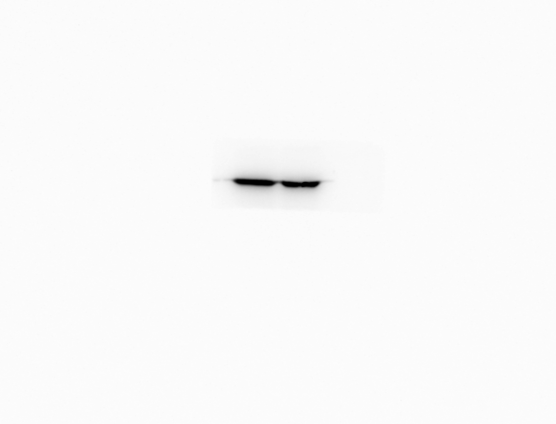


β-actin

Vector sh-Mettl1

Sptbn2

Vector sh-Mettl1

Figure 5G


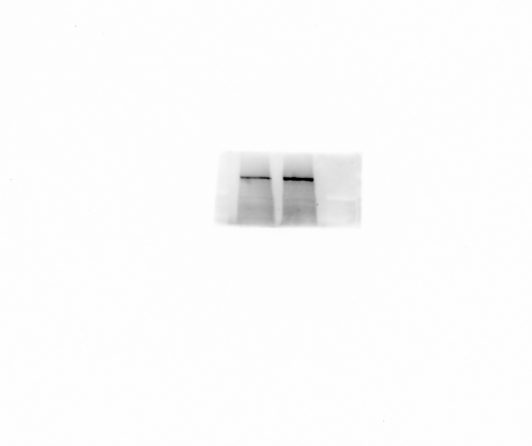

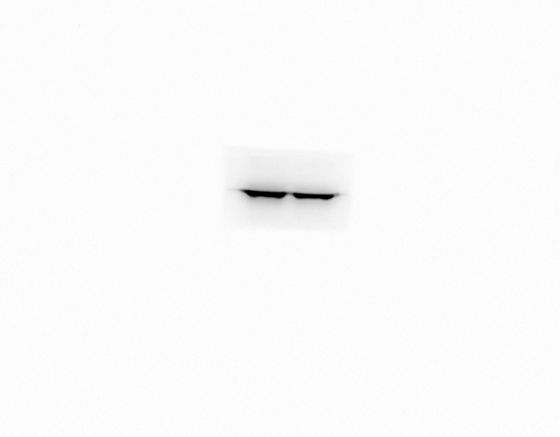


β-actin

Sptbn2

Vector OE-Mettl1

Vector OE-Mettl1

Figure 6A

Vector 1-sh-Sptbn2 2-sh-Sptbn2


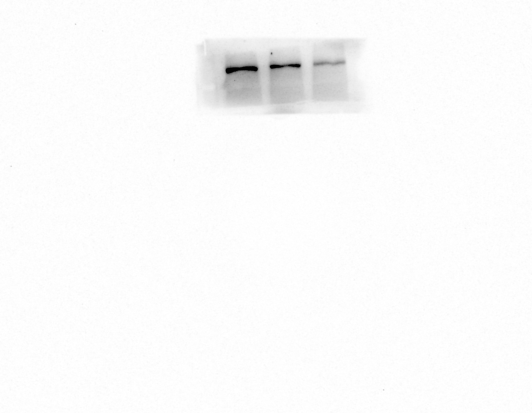

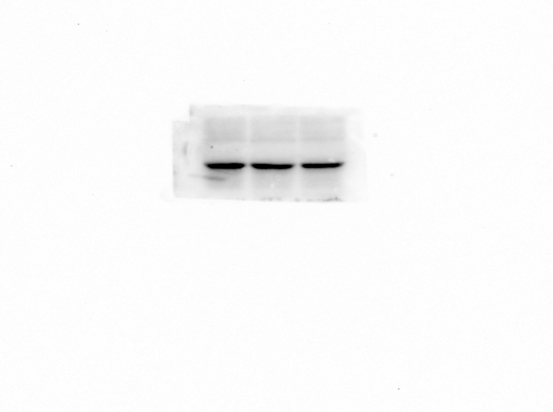


β-actin

Vector 1-sh-Sptbn2 2-sh-Sptbn2

Sptbn2

Figure 6G


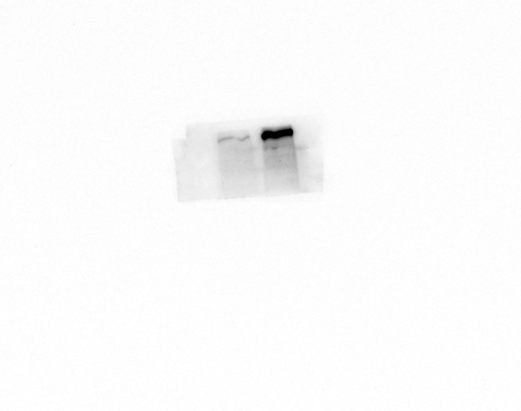

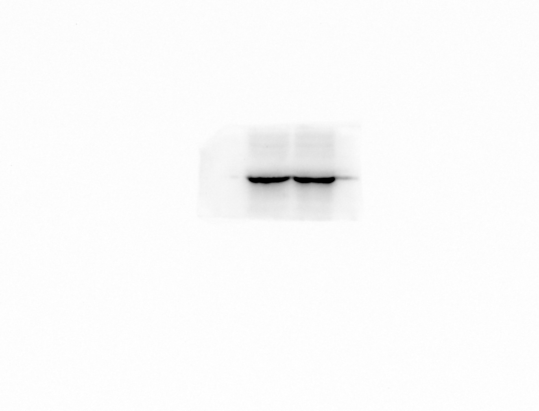


β-actin

Sptbn2

Vector OE-Sptbn2

Vector OE-Sptbn2

Figure 3A


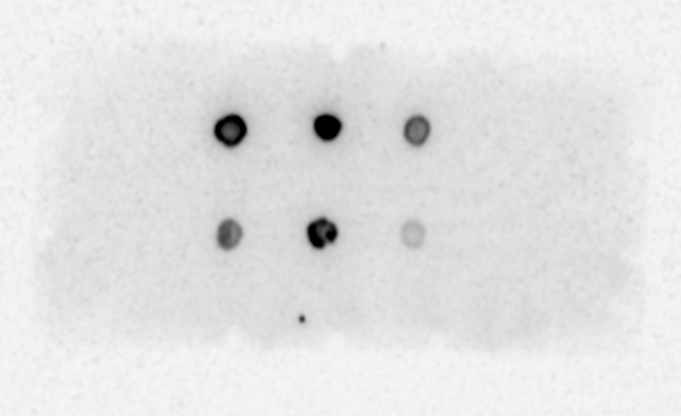


NSCs Neurons Astrocytes

200ng

100ng

m^7^G

Figure 3C


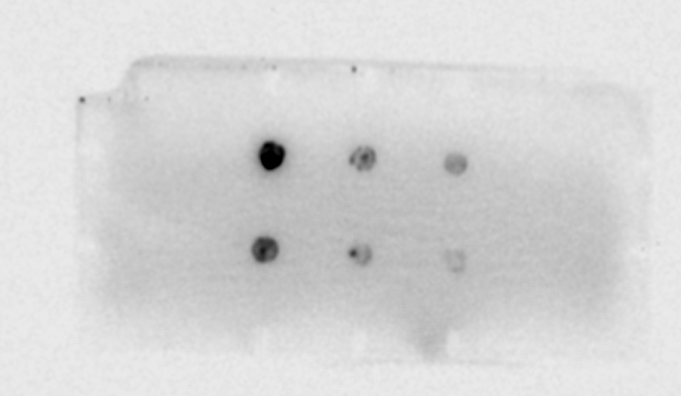


m^7^G

200ng

100ng

Vector 1-sh-Mettl1 2-sh-Mettl1

Figure 3E


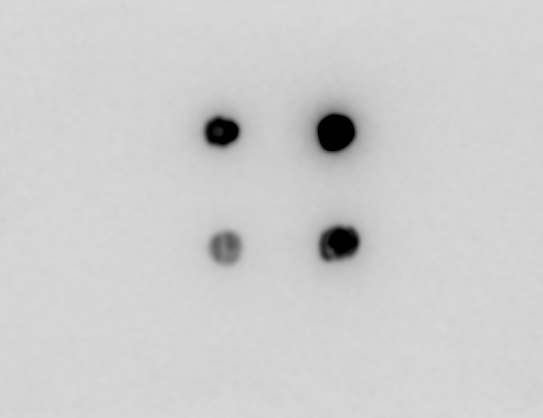


200ng

100ng

m^7^G

Vector OE-Mettl1
