# Supplementary material for: Mettl1-mediated internal m7G methylation of Sptbn2 mRNA elicits neurogenesis and anti-alzheimer’s disease
Source: Cell Biosci. 2023 Oct 1;13:183. doi: 10.1186/s13578-023-01131-2 (PMC10544167; doi:10.1186/s13578-023-01131-2)
Supplement: Supplementary file 2 — Supplementary Material 2 [file 13578_2023_1131_MOESM2_ESM.docx]

**Figure 1. The transcriptome landscape of internal m^7^G-modified mRNA during astrocytic differentiation of NSCs.**

(**A, B**) Bar plot chart showing the significant GO terms and KEGG analysis for NSCs and differentiated astrocytes mRNAs containing internal m^7^G. (**C**) Volcano plot of significantly altered internal mRNA m^7^G peaks in NSCs compared to astrocytes. (**D**) Scatter plot of RNA-Seq fold change and RIP-Seq fold change on 5'UTR. NSCs, neural stem cells.


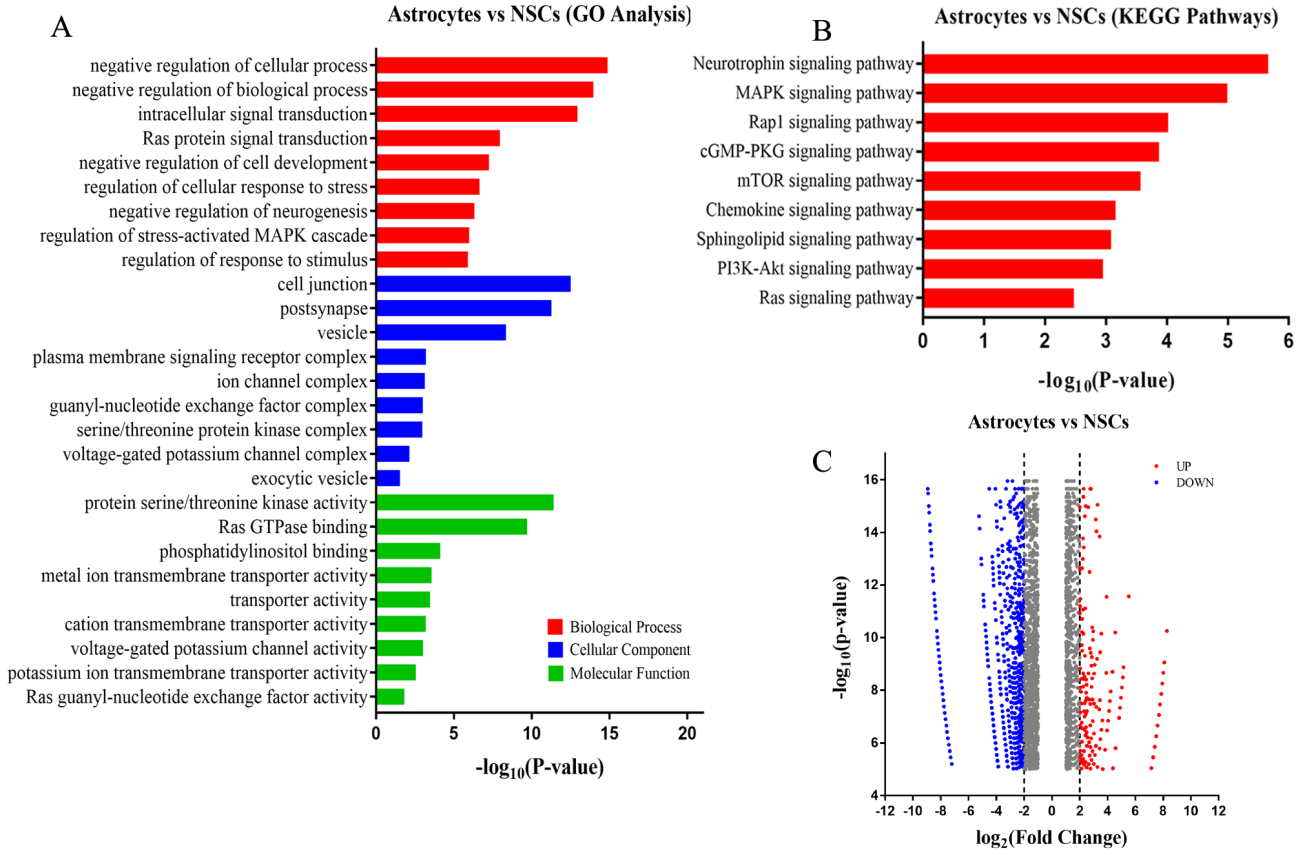


**D**

**
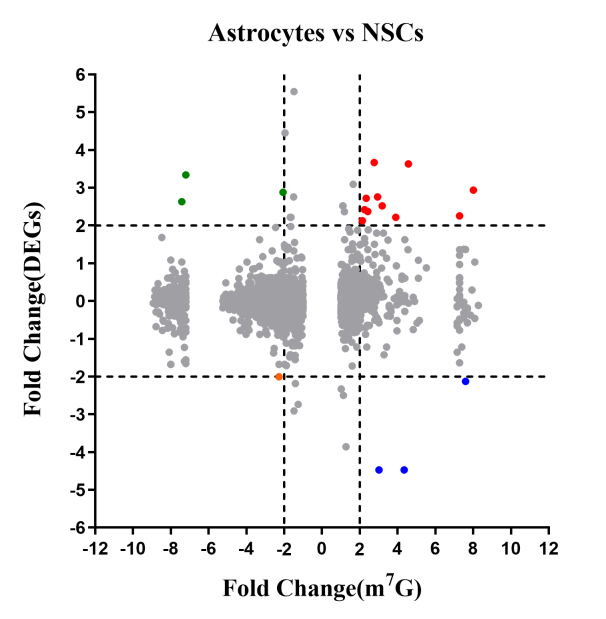
**
